# Programmatic Implementation of Contact Investigation in Eight African Countries

**DOI:** 10.3390/tropicalmed8010029

**Published:** 2022-12-30

**Authors:** Kobto G. Koura, Olivia B. Mbitikon, Attannon A. Fiogbé, Abdoul R. Ouédraogo, Albert Kuate Kuate, Aboubacar S. Magassouba, Alphazazi Soumana, Georges Hermana, Barnabé Gning, Mohammed F. Dogo, Monicah Andefa, Gisèle Badoum

**Affiliations:** 1 International Union Against Tuberculosis and Lung Disease, 75001 Paris, France; 2 COMUE Sorbonne Paris Cité, Faculté des Sciences Pharmaceutiques et Biologiques, Paris Descartes University, 75006 Paris, France; 3 National Tuberculosis Programme, Cotonou P.O. Box 321, Benin; 4 Service de Pneumologie, Centre National et Universitaire de Pneumo-Phitsiologie de Cotonou (CNHUPP/C), Cotonou P.O. Box 321, Benin; 5 Unité de Formation et de Recherche en Sciences de la Santé, Joseph Ki-Zerbo University, Ouagadougou P.O. Box 7021, Burkina Faso; 6 Service de Pneumologie, Centre Hospitalier et Universitaire de Tengandogo (CHU-T), Ouagadougou P.O. Box 104, Burkina Faso; 7 National Tuberculosis Programme, Yaounde P.O. Box 15656, Cameroon; 8 National Tuberculosis Programme, Conakry P.O. Box 634, Guinea; 9 National Tuberculosis Programme, Niamey P.O. Box 623, Niger; 10 National Tuberculosis Programme, Bangui P.O. Box 883, Central African Republic; 11 National Tuberculosis Programme, Dakar P.O. Box 4024, Senegal; 12 National Tuberculosis Programme, Lomé P.O. Box 2171, Togo; 13 Service de Pneumologie, Centre Hospitalier et Universitaire de Yalgado Ouédraogo (CNHU-YO), Ouagadougou P.O. Box 7022, Burkina Faso

**Keywords:** contact investigation, programmatic conditions, children under 5, PLHIV, Africa, TB treatment, TB preventive therapy

## Abstract

The objective was to implement CI under national tuberculosis programmatic conditions and to advocate for its scaling up. Contact investigation was implemented in 150 Basic Management Units identified across eight countries. The target populations (children <5 years and persons living with HIV (PLHIV)) were evaluated during home and clinic visits using standardized tools, clinical examinations and, according to each country, additional tests. Contacts with active TB received TB treatment and those eligible received TB preventive therapy (TPT). Data were collected each quarter using standardized forms. Meetings were organized with partners to share preliminary results and advocate for scaling up. From October 2020 to December 2021, 9049 home visits were performed. The proportions of children <5 years and PLHIV who were screened and diagnosed with active TB were, respectively, 2.6% and 10.1%. Ninety-three percent of children <5 years and 98% of PLHIV living at home received TPT or TB treatment, respectively. The scale-up for contact investigation partially or at national level in 2022 was effective in six of the eight countries included in the project. These results indicate that CI is feasible under programmatic conditions within the National TB Programs of African countries.

## 1. Introduction

Contact investigation (CI) is defined by the World Health Organization (WHO) as the systematic identification of people with previously undiagnosed tuberculosis (TB) disease and TB infection among the contacts of an index TB patient in the household and in comparable settings in which transmission occurs. It consists of the identification, clinical evaluation and/or testing and provision of appropriate anti-TB therapy (for people with confirmed TB) or TB preventive treatment (for those without TB disease and eligible for such therapy) [1].

Several systematic reviews have evaluated the yield of contact investigation in low- and middle-income countries. They found that the percentage of contacts screened who were diagnosed with active TB (bacteriologically confirmed, or clinically diagnosed) varied from 2.9 to 4.5% [2,3,4,5]. They concluded that CI increased case detection, reduced mortality, decreased the community prevalence of TB and resulted in a high yield of co-prevalent, incident and latent TB infection among contacts [5].

Despite the benefits of this intervention, and although this intervention has been recommended for several years, it is not fully implemented and is also poorly documented in low- and middle-income settings in programmatic conditions. From 2015 to 2019, The International Union Against Tuberculosis and Lung Disease (The Union) implemented operational research, the TITI project, in four countries (Benin, Burkina Faso, Cameroon and the Central African Republic) and demonstrated that CI could be integrated with the programmatic activities of the national tuberculosis programme (NTP) with a few additional resources [6].

The TITI project findings led The Union, in collaboration with NTPs, to include the intervention in the first component of a new project called “Contributing to the Elimination of Tuberculosis in Africa (CETA) project”. The objectives of the first component of this project were to implement CI under programmatic conditions and to advocate for its scaling up at national level. This paper shares the steps of the implementation of CI under programmatic conditions, the first results of this implementation, the status of scaling up in the countries, the lessons learned and the challenges and opportunities to ensure the successful scale-up of CI at national level in high TB burden countries.

## 2. Materials and Methods

### 2.1. Study Approach

The CETA project aims to Contribute to the Elimination of Tuberculosis in Africa by 2035. The project supports NTPs with TB screening and prevention, improving healthcare delivery and strengthening the governance of the NTPs. The project creates a network of African experts who support each other and have ongoing access to the expertise of The Union, through workshops, courses, conferences and online resources, with the aim of improving the care offered to people with or at risk of TB.

The first component aims to improve TB screening and prevention with a particular focus on improving access to care for vulnerable populations. Using contact investigation, the project assists with the early detection of active TB, allowing those diagnosed to be treated quickly, and ensuring preventive therapy for any close and eligible contacts in order to stop the spread of the disease.

The project was implemented in the countries by The Union in collaboration with the NTPs.

### 2.2. Study Site and Population

**Figure 1:**
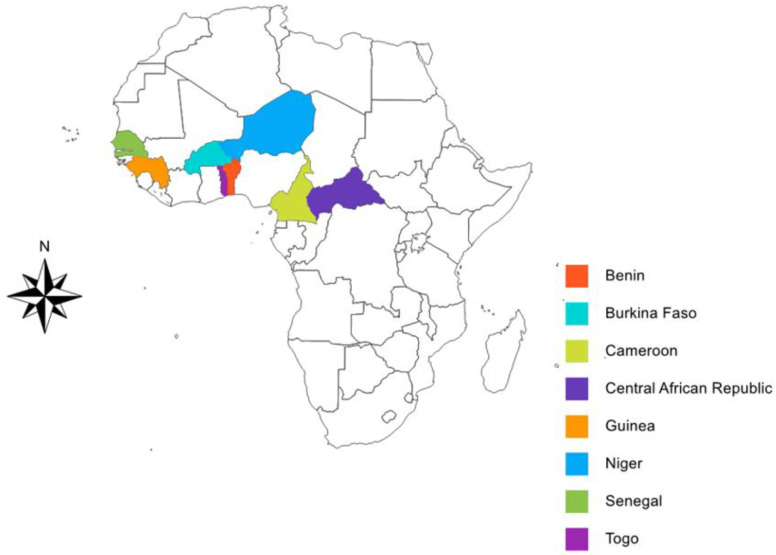
Countries with study sites included in this paper.

The CI was implemented under programmatic conditions in eight African countries: Benin, Burkina Faso, Cameroon, Guinea, Niger, the Central African Republic, Senegal and Togo (see Figure 1). The software “Cartes & Données—© Articque” was used to develop the study map.

One hundred and fifty (150) Basic Management Units (BMU) were identified across the eight countries based on the following criteria: (1) number of pulmonary bacteriologically confirmed TB cases (PTB+) diagnosed in the previous year in each BMU (at least 30); (2) geographic and security accessibility; (3) absence of support from another partner for the same activities.

The study population was composed of index cases and contacts. Index cases are usually defined as a person of any age with new or recurrent TB in a specific household or comparable setting in which others may have been exposed [1]. In this current study, the index case was defined as a person with new or recurrent PTB+. A contact is usually defined as any person who has been exposed to a person with TB disease [1]. The contacts in this current study were children <5 years and people living with HIV (PLHIV). Children <5 years living with a PTB+ have a higher risk of being infected and of developing TB [2,7]. Because of their immunosuppression, PLHIV are at high risk of progressing from TB infection to TB disease either by reactivation of an old TB infection or by rapid progression of a recent TB infection [8,9]. The risk of developing TB in PLHIV is 20 times higher than the risk in the general population.

The index case and their contacts were recruited between October 2020 and September 2021. The contacts were followed up until March 2022.

### 2.3. Data Collection

#### 2.3.1. Kick-Off Meeting

A kick-off meeting took place virtually on 30 July 2020. The Union staff, the eight NTP Managers and their teams and the partners (Alliance Côte d’Ivoire, DRAF TB, Global Fund, WHO Global TB Program, WHO TDR) attended this meeting. The objectives of the project were discussed as were the strategies for implementing CI according to the target populations. The standardized tools for implementation as well as for monitoring this intervention were developed and validated. Seven tools were developed for data collection: a home visit workbook, an appointment form, a chest X-ray request form, an electronic register, a contact’s stamp, a contact’s card and a quarterly report.

#### 2.3.2. Tools Adaptation, National Training

After the kick-off meeting, each country adapted the developed tools.

An initial 3-day training course was organized in each country, and a total of 415 medical doctors, nurses and community healthcare workers (CHW) were trained. During each training session, the participants were informed about the objectives of CI. They were also trained on the procedures to implement CI on children <5 years and PLHIV. The responsibilities and roles of the participants were discussed. They were also trained on the use of the tools for this intervention.

#### 2.3.3. Procedures for CI

All patients newly diagnosed with PTB+ received detailed verbal information about the intervention and an appointment for a home visit was arranged within 3 days of the interview.

**Figure 2:**
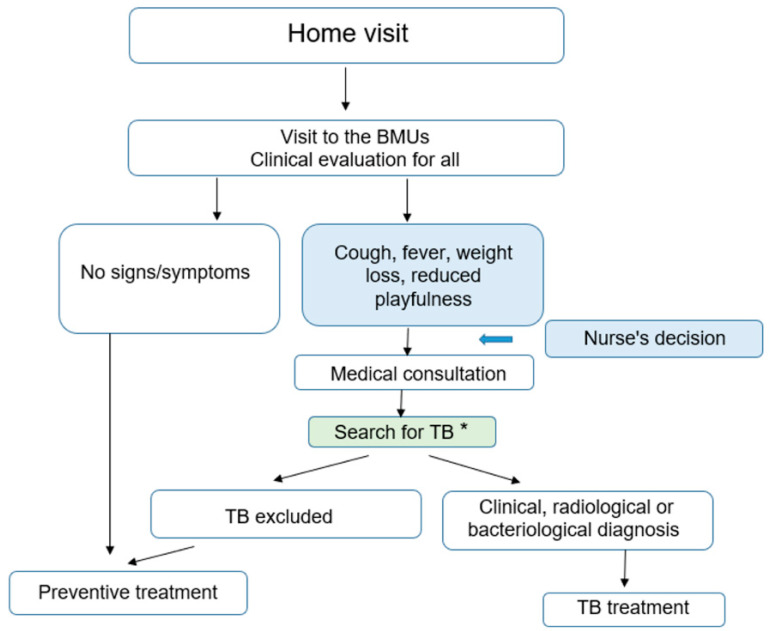
Screening algorithm used for children under 5 years. * the following additional tests: (1) Chest X-Ray; (2) Sputum specimens for Smear and/or Xpert MTB/RIF depending on the country.

**Figure 3:**
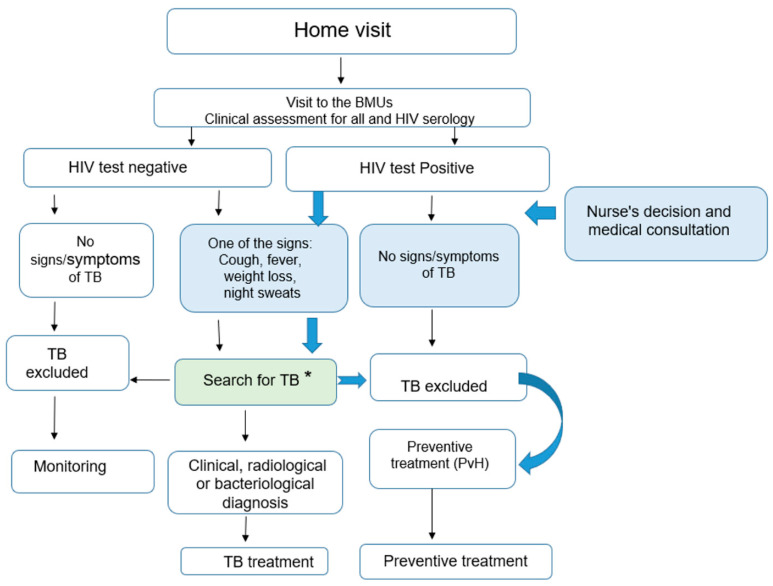
Screening algorithm used for adults and adolescents. * the following additional tests: (1) Chest X-Ray; (2) Sputum specimens for Smear and/or Xpert MTB/RIF depending on the country.

The home visits were performed by a nurse and/or the CHW. The transport costs for the home visit were supported by the project. The home visit workbook was used to list all contacts (children and adults) living in the household. Their ages and all the symptoms suggestive of TB of any duration during the previous 4 weeks were reported: cough, fever, weight loss (or reduced appetite for children under 5 years), asthenia (or reduced playfulness for children under 5 years). For contacts with signs and/or symptoms suggesting TB a chest X-ray (CXR) request form was given. The costs for CXR were not covered either by the NTP or by this programmatic project in 7 out the 8 countries, except in Burkina Faso where the CXR was free of charge for children <5 years. An appointment form for a review of the Basic Management Units (BMUs) 2–3 days after the home visit was given to the contacts. At the BMUs, two scenarios occurred, one for children <5 years and another one for adults and adolescents:

- Children <5 years were clinically re-evaluated and the nurse read the results of the CXR when available. The nurse referred children with any radiographic abnormality or with any signs or symptoms suggestive of TB to a doctor. The latter conducted a full clinical examination, re-interpreted the CXR and requested a collection of sputum specimens for smear microscopy and Xpert MTB/RIF testing. The decision to administer anti-tuberculosis treatment was then taken by the doctor on the basis of this assessment. Children without evidence of active disease after this evaluation were immediately started on TB preventive treatment (see Figure 2).

- Adults and adolescents were re-evaluated and the nurse read the results of the CXR. HIV tests were performed (see Figure 3):
  ○ If the HIV test was negative and there were no signs and symptoms of TB, TB was excluded, and no preventive therapy was provided to these contacts. They were informed about the symptoms and signs of TB and requested to come back to the BMU if any of these symptoms and signs occurred. For the other adults and adolescents, if there were any signs or symptoms suggestive of TB with any radiographic abnormality, the contacts were referred to a doctor who conducted a complete clinical examination, re-interpreted the CXR and requested sputum specimen collection for smear microscopy and Xpert MTB/RIF testing. The decision to administer anti-tuberculosis treatment was then taken by the doctor on the basis of this assessment.
  ○ In contrast, if the HIV Test was positive and there were no signs and symptoms suggestive of TB and radiography was normal, TB was excluded and the PLHIV contact received TB preventive treatment. If there were any signs or symptoms suggestive of TB with any radiographic abnormality the contacts were referred to a doctor who conducted a full clinical examination, re-interpreted the CXR and requested a collection of sputum specimens for smear microscopy and Xpert MTB/RIF testing. The decision to administer anti-tuberculosis treatment was then taken by the doctor on the basis of this assessment.

#### 2.3.4. Tuberculosis Treatment and Preventive Treatment

All drugs used for TB treatment as well as for TB preventive treatment were pre-qualified and provided through the Global Drug Facility mechanism. New contacts with pulmonary TB received a regimen consisting of an intensive phase of 2 months of isoniazid (INH), rifampin (RIF), pyrazinamide (PZA) and ethambutol (EMB) followed by a continuation phase of 4 months of INH and RIF.

For TB preventive therapy for children <5 years, the 3-month rifampicin-isoniazid regimen (3RH) was given, except in Benin, where six months of isoniazid alone (6H) was administered. In all countries, eligible HIV-infected contacts were given 6H because of possible drug–drug interactions (rifampicin) with antiretroviral treatment.

### 2.4. Data Analysis

For this programmatic implementation, aggregated data were collected using the standardized forms designed for the project and validated during the kick-off meeting. Each quarter, the nurses of the BMUs sent their screening and their treatment outcome reports to the central level of the NTP. These reports were compiled by BMU, region, quarter and year using Microsoft Excel 2016 for Windows. A monitoring file was also developed for the project using Microsoft Excel 2016 for Windows. This monitoring file compiled the quarterly data provided by the eight countries. The BMUs were regularly supervised by the central level. Calls were also made to correct all discrepant data. Statistical analyses and elaboration of the tables were performed using Microsoft Excel 2016 for Windows.

### 2.5. Ethics

As the study was implemented under programmatic conditions, formal ethics approval was deemed not to be necessary. No individual data were collected. Only aggregated information was collected by those providing care for TB patients and their contacts and no individual identifiers were provided to individuals outside the health service.

## 3. Results

### 3.1. Proportion of Home Visits Performed among Index Cases

From the last quarter of 2020 to the last quarter of 2021, a total of 21,945 PTB+ patients were diagnosed through 150 BMUs. Nine thousand and forty-nine (41%) of these index cases were visited at home (Table 1). This proportion differed by country and ranged from 23% in the Central African Republic to 87% in Togo.

### 3.2. Proportion of Contacts Living at Home and on Preventive Treatment or TB Treatment

#### 3.2.1. Children under 5 Years

A total of 14,290 children <5 years were living at home across the 150 BMUs. Among these screened children: (1) 2.6% [(378 ÷ 14,290) × 100] were diagnosed with active TB (bacteriologically confirmed, or clinically diagnosed) and started TB Treatment; (2) 91% [(12,983 ÷ 14,290) × 100] started TB preventive treatment after the exclusion of TB.

The proportion of children <5 years on TPT or TB Treatment was 93% [(12,983 + 378) ÷ 14,290]. This proportion was only found for index cases that had home visits. This proportion differed by country, and ranged from 67% in Burkina Faso to 100% in Benin, Cameroon, Guinea, the CAR and Togo (Table 2).

#### 3.2.2. PLHIV

A total of 287 PLHIV were living at home across the 150 BMUs. Among these screened PLHIV: (1) 10% [(29 ÷ 287) × 100] were diagnosed with active TB (bacteriologically confirmed, or clinically diagnosed) and started TB Treatment; (2) 88% [(252 ÷ 287) × 100] started TB preventive treatment after the exclusion of TB.

The proportion of PLHIV on TPT or TB Treatment was 98% [(252 + 29) ÷ 287]. This proportion was only found for index cases that had home visit. This proportion differed by country and ranged from 50% in Guinea to 100% in Burkina Faso, Cameroon, Niger, the CAR and Senegal (Table 3).

### 3.3. Ratio of the Number of Contacts Living at Home to the Number of Index Cases

The ratio of the number of children <5 years living at home to the number of index cases was 0.65 and ranged from 0.41 in Togo to 1.12 in Burkina Faso (Table 4).

The ratio of the number of PLHIV living at home to the number of index cases was 0.013 and ranged from 0.001 in Guinea to 0.033 in Cameroon (Table 5).

### 3.4. Scale Up

Table 6 provides the status of the scale-up of the intervention at national level at the beginning of 2022. In Benin, the implementation of contact investigation at national level was already effective only in children <5 years. The scale-up for PLHIV contacts is not yet happening.

The scale-up at national level of the strategy described in this project is ongoing in two countries: Burkina Faso and Cameroon under the Global Fund grants. A partial scale-up is ongoing under Global Fund grants in two countries (Guinea and Niger) and under The Union grant in one country (Togo). Two countries (CAR and Senegal) have not yet scaled up the intervention either partially or at national level.

## 4. Discussion

There were several key findings from this project. First, at the programmatic level, an impressive 41% of the index cases in eight African countries were visited at home. Second, for children <5 years living with a person with PTB+ we found that 2.6% were diagnosed with active TB, a high proportion started TPT (91%), and the ratio of these children to the number of index cases was 0.65. Third, for PLHIV living with a person with PTB+ we found that 10.1% were diagnosed with active TB, a high proportion started TPT (88%), and the ratio of these PLHIV to the number of index cases was 0.013. Finally, the scale-up for contact investigation partially or at national level in 2022 was effective in six of the eight countries included in the project.

The proportion of index cases visited at home in this study (41%) was higher than the proportion of index cases visited at home in our previous TITI study in four African countries (1095 ÷ 4303 = 25%). The TITI project was an operational research study with certain inclusion criteria that might have decreased the proportion of contacts able to have home visits: these inclusion criteria were: (1) having smear-positive TB; (2) having rifampicin susceptibility, either presumptive or confirmed on Xpert MTB/RIF (Cepheid, Sunnyvale, CA, USA); (3) being aged 15 years; (4) living within 5 km of the clinic; (5) having resided at the place of residence for 3 months; and (6) living at home with at least one child <5 years. This proportion of index cases visited at home differed by country. Four countries had the lowest proportion (the CAR, Guinea, Senegal and Cameroon). Two main reasons could explain these findings. There is a repeated turnover of the staff in these countries compared to the other countries, and at each new appointment it is a perpetual beginning with staff needing to be trained. The second and non-negligible reason is the fact that the HCW were involved in several activities at the BMUs. They prioritize these activities to the detriment of the home visits of the index cases and the field visit revealed that the main reason was the low incentive provided by the project.

The proportion of children <5 years with TB (2.6%) found in this project was inferior with the proportion described in our previous TITI project (2.8%) as well as in several other projects [2,3,4,5,6,10]. This proportion was higher than the one described in Gambia and in Pakistan [11,12]. The high proportion of children <5 years started on TPT (91%) was similar to the proportion of our previous TITI project [6]. This proportion was higher than that reported in other studies in Africa: 13.2% in South Africa, 64% in Uganda and 83% in Guinea Bissau [13,14,15]. The higher proportion observed in our programmatic project suggests a better adherence to the HCW’s recommendations for tuberculosis prevention. This better adherence could be explained by the persuasiveness skills of the health care workers and by the culture of the region. Regarding the culture, Iwelunmor et al. have shown that, for different conditions/behaviours/experiences, culture remains a key determinant for effectively examining the impact on health and thus framing solutions [16]. Although the ratio of children to the number of index cases (0.65) was inferior to the ratio we found in our previous study (0.8), it could be used for estimating the drug needs of countries and for ordering these drugs. However, as the proportion of index cases visited at home is low, this ratio should be updated each year according to the results of the previous year’s contact investigation activities. The best estimation of this ratio could be obtained only if at least 80% of index cases were visited at home.

To our knowledge, this is the first study that implemented CI for PLHIV living at home with bacteriologically confirmed TB patients. The number of PLHIV at home was very low. The main reason reported by the staff was that TB patients, due to stigmatization, did not want to talk about HIV at home during a home visit. For the PLHIV screened, we found that the proportion diagnosed with TB was high. This proportion has certainly been underestimated, as home visits have been performed for only 41% of index cases. The PLHIV, due to their immunosuppression, represent a high-risk population for TB. They are at high risk of progression from TB infection to TB disease either through the reactivation of an old TB infection or through the rapid progression of a recent TB infection [8,9]. The risk of developing tuberculosis in PLHIV is 20 times higher than the risk in the general population. The ratio of PLHIV to the number of index cases could be used as a starting point for the estimation of the drug needs of countries and for ordering the appropriate drugs. However, this ratio needs to be updated each year according to the results of the previous year’s contact investigation activities. The best estimation of this ratio could be obtained only if at least 80% of index cases were visited at home.

There were several strengths and limitations of this project. The study was carried out at national level and under routine programmatic conditions and therefore is representative of what is happening on the ground. The findings of this project are also consistent with reports and publications from several other projects. There were, however, several limitations. Only aggregate data were collected, and thus we were not able to assess what socio-economic and clinical factors were associated with being able to start TB treatment or TB preventive therapy.

The implementation of the project has enabled us to identify several challenges for which there are potential solutions. First, despite the training, some countries noted a low level of involvement and proficiency of the CHW. Some countries also experienced a high turnover of trained staff. Second, there were difficulties in effectively referring children to be evaluated for TB disease before the initiation of TPT and this was especially the case if the children were healthy. There are particular difficulties in accessing health services for populations who live in areas considered unsafe (some areas in Niger and the CAR). Third, the project highlighted the lack of coordination between child health and TB services as well as the refusal of some parents to agree to TPT for their well child. Finally, the COVID-19 pandemic reduced attendance at clinics for referral assessment or follow-up.

In order to scale up this project activity, several actions are necessary that can be quickly implemented. The national policy on CHW should be reviewed and updated to include TB contact investigation in the package of the activities that can be undertaken by CHW. The capacity of healthcare providers to detect and manage childhood TB as well as TB in PLHIV needs strengthening with better collaboration between the NTP and paediatric services. The development of a communication and advocacy plan is required to improve community education and engagement, and financing, including for home visit costs, could be included in Global Fund grants. The implementation of a simple clinical algorithm (usable in communities by healthcare workers) would help the TB screening process. We also need to embrace advances in digital technology, including portable and ultra-portable digital chest radiography with computer-aided TB diagnosis, which will greatly enhance the efficiency and effectiveness of screening for TB. The current project focused on CI for children under 5 years and PLHIV living with PTB+. However, it is important to extend these activities for all the contacts of PTB+ whatever their age. The lessons learnt for this project could be used.

## 5. Conclusions

The results of this project indicate that CI is feasible under routine programmatic conditions within the NTPs of African countries. The lessons learned through this implementation are currently being used for the scale-up phase. The scaling up financed by the Global Fund and The Union in some countries reflects the good collaboration between NTPs and international partners.

## Figures and Tables

**Table 1:** Proportion of home visits performed among index cases, Q4 2020 to Q4 2021.

Country	N Index Cases	N Index Cases Visited at Home Visit	Ratio
Benin	813	583	72%
Burkina Faso	1525	1261	83%
Cameroon	2114	660	31%
Guinea	4613	1351	29%
Niger	2382	1673	70%
Central African Republic	5086	1145	23%
Senegal	4100	1231	30%
Togo	1312	1145	87%
Total	21,945	9049	41%

**Table 2:** Proportion of children under 5 years living at home and on preventive treatment or TB treatment.

Country	<5 Years Living at Home	<5 Years on TPT	<5 Years on TB Treatment	Proportion of children <5 Years on TPT or TB Treatment
Benin	564	560	4	100%
Burkina Faso	1713	1143	13	67%
Cameroon	956	936	20	100%
Guinea	3884	3677	207	100%
Niger	1885	1542	92	87%
CAR ^1^	3043	3022	21	100%
Senegal	1709	1571	17	93%
Togo	536	532	4	100%
Total	14,290	12983	378	93%

^1^ Central African Republic.

**Table 3:** Proportion of PLHIV living at home and on preventive treatment or TB treatment.

Country	PLHIV Living at Home	PLHIV on TPT	PLHIV on TB Treatment	Proportion of PLHIV on TPT or TB Treatment
Benin	20	13	6	95%
Burkina Faso	22	13	9	100%
Cameroon	70	64	6	100%
Guinea	6	3	0	50%
Niger	4	4	0	100%
CAR ^1^	104	99	5	100%
Senegal	11	10	1	100%
Togo	50	46	2	96%
Total	287	252	29	98%

^1^ Central African Republic.

**Table 4:** Ratio of the number of children under five years living at home to the number of index cases.

Country	N Index Cases	<5 Years Living at Home	Ratio
Benin	813	564	0.69
Burkina Faso	1525	1713	1.12
Cameroon	2114	956	0.45
Guinea	4613	3884	0.84
Niger	2382	1885	0.79
CAR ^1^	5086	3043	0.60
Senegal	4100	1709	0.42
Togo	1313	533	0.41
Total	21,946	14,287	0.65

^1^ Central African Republic.

**Table 5:** Ratio of the number of PLHIV living at home to the number of index cases.

Country	N Index Cases	PLVIH Living at Home	Ratio
Benin	813	20	0.025
Burkina Faso	1525	22	0.014
Cameroon	2114	70	0.033
Guinea	4613	6	0.001
Niger	2382	4	0.002
CAR ^1^	5086	104	0.020
Senegal	4100	11	0.003
Togo	1313	52	0.040
Total	21,946	289	0.013

^1^ Central African Republic.

**Table 6:** Scale-up of contact investigation at national level.

Country	Current Situation
Benin	Scale-up is done under GF funding for children under 5 years only.
Burkina Faso	National scale-up ongoing under the Global Fund funding. The GF covers 10/13 regions in 2022 and 13/13 regions in 2023. The Union provided funds for the multiplication of the tools.
Cameroon	Progressive national scale-up under Global Fund funding (NFM3). In 2022, 6 regions out of 10 are already covered. The four other regions will be covered in 2023.
Guinea	Partial scale-up under the Global Fund grant
Niger	Partial scale-up started in 2022 (training of stakeholders and conducting investigations around the cases on the new Global fund grant 2022–2024 and COVID-19 RM contingency plan).
Togo	Partial scale-up through the Union’s funds
CAR ^1^	Scale-up has not yet started
Senegal	Scale-up planned under the Global Fund grant but has not yet started

^1^ Central African Republic.

## Data Availability

The data that support the findings of the study are available from one of the first authors (K.K.G.) upon reasonable request.
